# Spontaneous Spinal Epidural Hematoma Secondary to Rivaroxaban Use in a Patient With Paroxysmal Atrial Fibrillation

**DOI:** 10.7759/cureus.10417

**Published:** 2020-09-12

**Authors:** Armaghan Raeouf, Siddarth Goyal, Nicole Van Horne, Jeremy Traylor

**Affiliations:** 1 Department of Medicine, Lake Erie College of Osteopathic Medicine, Erie, USA; 2 Department of Emergency Medicine, Grandview Medical Center, Dayton, USA

**Keywords:** spinal epidural hematoma, atrial fibrillation, xarelto, rivaroxaban, low back pain

## Abstract

A spontaneous spinal epidural hematoma (SSEH) is a rare condition of intraspinal bleeding in the epidural space. It is often associated with disorders of anticoagulation, intraspinal tumors, vascular malformations, and pertinent to this case, anticoagulation therapy. This surgical emergency requires early diagnosis and management in order to minimize permanent neurologic deficits.

We report the case of a 72-year-old female with a past medical history of paroxysmal atrial fibrillation treated with rivaroxaban who presented to the emergency department with acute-onset, midline, lower back pain with no known trauma or injury to the area. At the time of emergency department (ED) admission, the patient was fully ambulatory and alert and oriented. However, within hours, she developed bilateral lower extremity motor paralysis and diminished sensation with urinary and bowel incontinence.

SSEHs are rare, progressive neurologic emergencies that can present with non-specific lower back pain. This condition presents a diagnostic challenge that can result in permanent neurologic defects if not recognized early. Emergency physicians regularly encounter patients with both acute lower back pain and atrial fibrillation. This case can contribute to the possibility of SSEH to a differential diagnosis.

## Introduction

Spontaneous spinal epidural hematomas (SSEHs) refer to non-traumatic and non-iatrogenic intraspinal bleeding above the dural sac. It is often associated with disorders of anticoagulation, intraspinal tumors, vascular malformations, and pertinent to this case, anticoagulation therapy [1,2]. Rivaroxaban is a Factor Xa inhibitor oral anticoagulant that is becoming more ubiquitous over heparin or vitamin K antagonists in the treatment of thromboembolic disorders such as atrial fibrillation [3].

Spinal epidural hematomas have an estimated annual incidence of 0.1 in 100,000 [4,5]. However, spontaneous cases are rare phenomena as roughly 500 cases have been reported since 1869 [6]. Patient clinical presentation will vary. The hematoma can be painless initially, and subsequently progress into severe, localized back pain due to the amounting dural strain [3]. If left untreated, SSEHs cause axonal damage similar to that of traumatic spinal cord injuries [7]. As expected, favorable outcomes have been shown with early diagnosis and prompt surgical intervention. Clinical suspicion can be confirmed with MRI. First-line management includes early surgical decompression via laminectomy [8,9].

## Case presentation

A 72-year-old female with a past medical history of paroxysmal atrial fibrillation on rivaroxaban, tachycardia-bradycardia syndrome, and chronic left prosthetic knee joint infection on antibiotics presented to the ED with a chief complaint of midline, lower back pain. She admitted to falling asleep the night before on a recliner and awakening with midline lower back pain that had been constant since her arrival to the ED. Her pain did not radiate anywhere. Patient reported a history of chronic low back pain, but not to the intensity that she felt that morning. She denied any trauma or injury to the area. Patient’s pain was exacerbated upon flexing and extending her spine. Sitting up and walking relieved her pain. She attempted to manage her discomfort with her home medications, which included percocet, robaxin, and flexeril, four hours prior to arrival, with no relief. On initial evaluation, the patient denied numbness of the lower extremities, urinary incontinence, bowel incontinence, difficulty with ambulation, chest pain, shortness of breath, nausea, vomiting, headaches, or changes in vision.

Presenting vital signs were 187/121mmHg, 99 bpm, 12 breaths/min, SpO2 100%, and the patient was afebrile. Upon physical examination, the patient was mildly distressed and anxious, but alert and oriented x3. Her back showed no signs of ecchymosis, rash, or laceration. Active range of motion and sensation of the spine and lower extremities were intact. She had 5/5 muscle strength in upper and lower extremities and patellar reflexes were intact. No abnormalities were noted in her gait. Significant laboratory findings are shown in Table 1.

**Table 1:** Significant laboratory findings AST, aspartate aminotransferase; ALP, alkaline phosphatase; WBC, white blood cell; INR, international normalized ratio

Significant Laboratory Findings					
Test		Result		Normal Range	
Complete Metabolic Panel					
Lactic acid		2.2		0.4-2.0 mmol/L	
Albumin		2.5		3.5-8.2 g/dL	
AST		54		15-37 U/L	
ALP		227		45-117 U/L	
Bilirubin, direct		0.48		0-0.2 mg/dL	
Hematology					
WBC		13.1		4.0-10.5 x 10^3^/uL	
Hemoglobin		11.5		12.1-15.8 g/dL	
Coagulation Studies					
INR		1.8		2.0-3.0	

**Figure 1:**
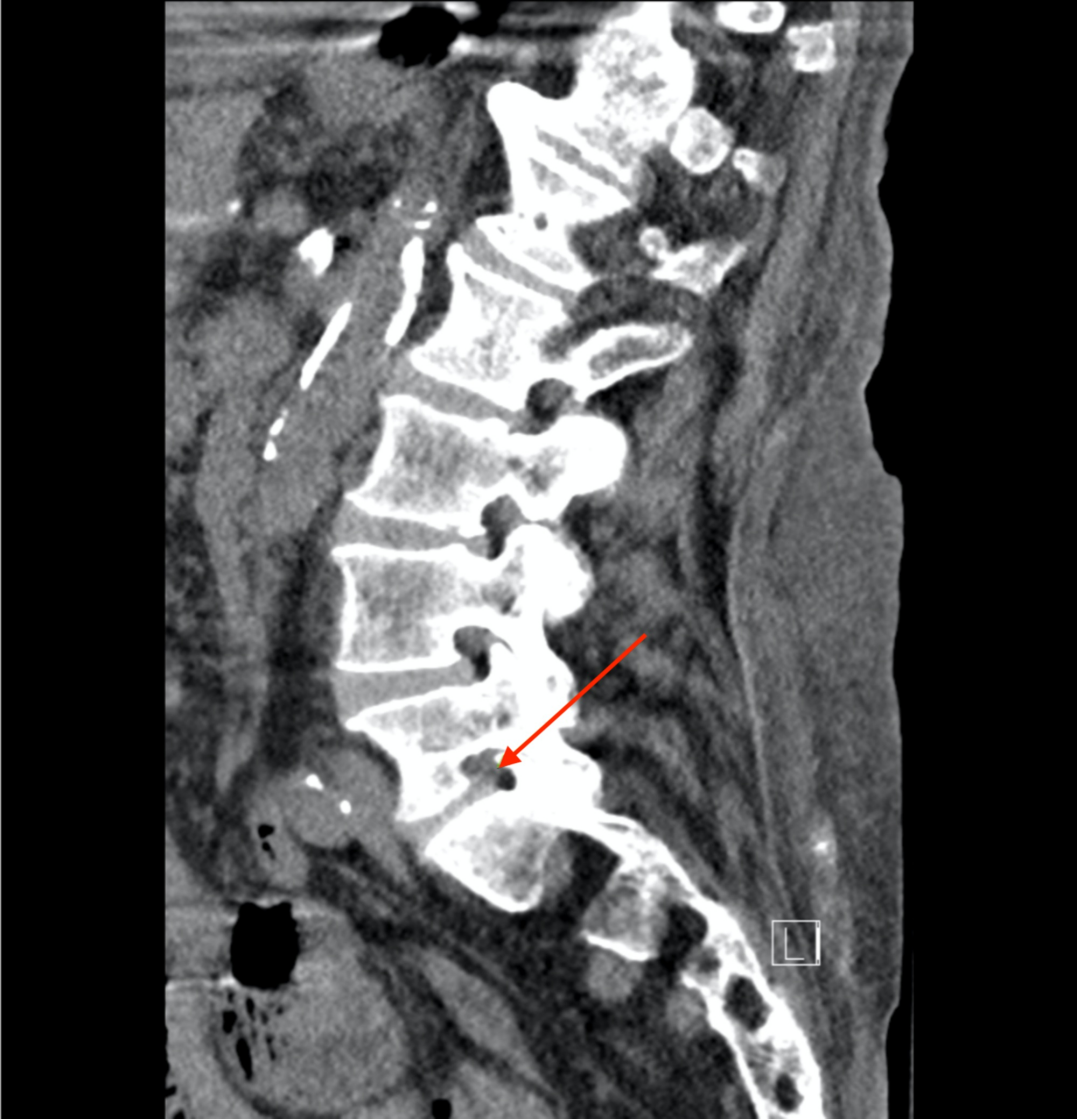
Lumbar CT without contrast The red arrow points to neuroforaminal narrowing at L5-S1 on the left

**Figure 2:**
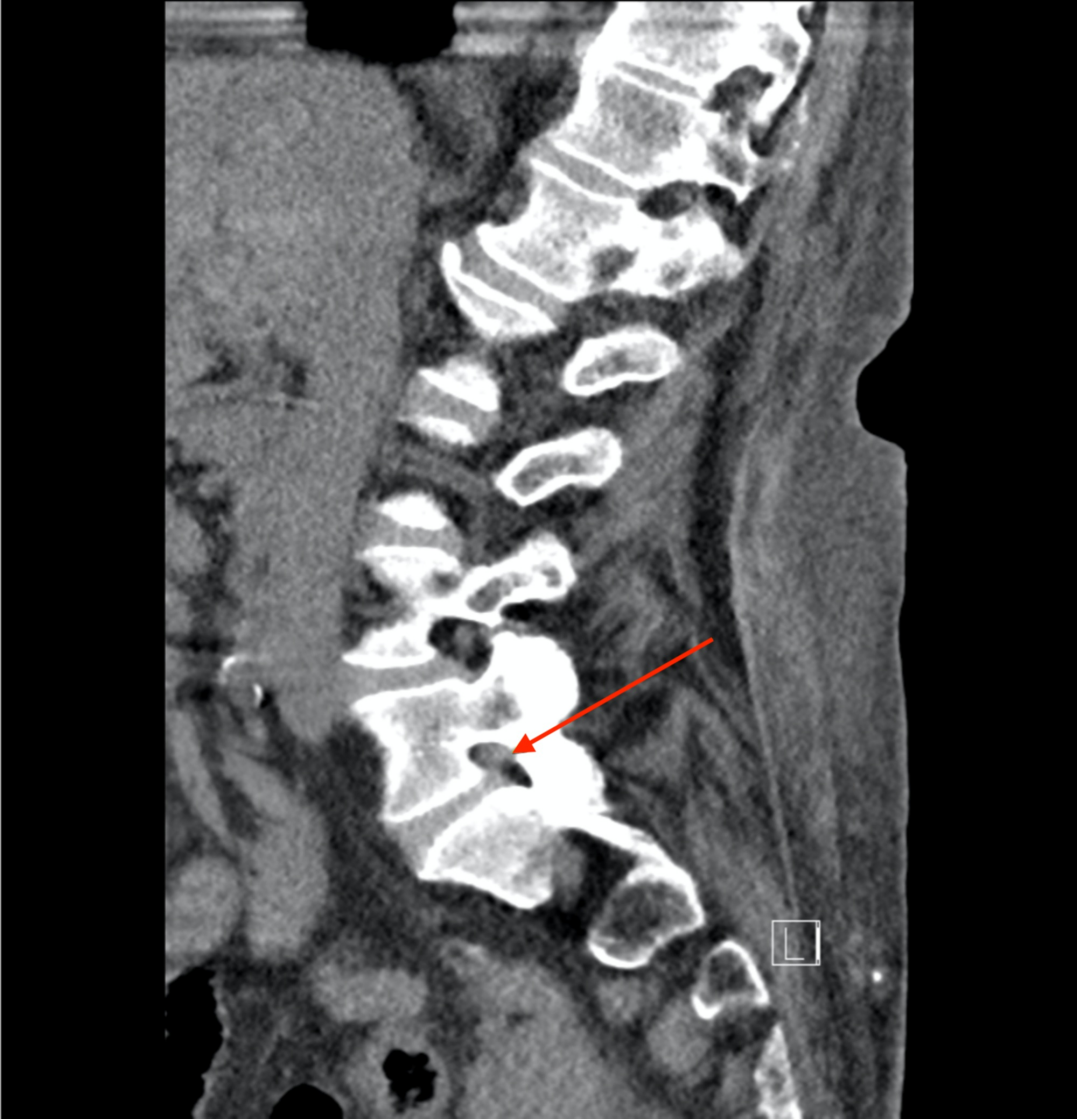
Lumbar CT without contrast The red arrow points to neuroforaminal narrowing at L5-S1 on the right

Patient was taken to radiology for a lumbar CT without contrast which identified moderate bilateral neuroforaminal narrowing at L5-S1 (Figures 1, 2).

**Figure 3:**
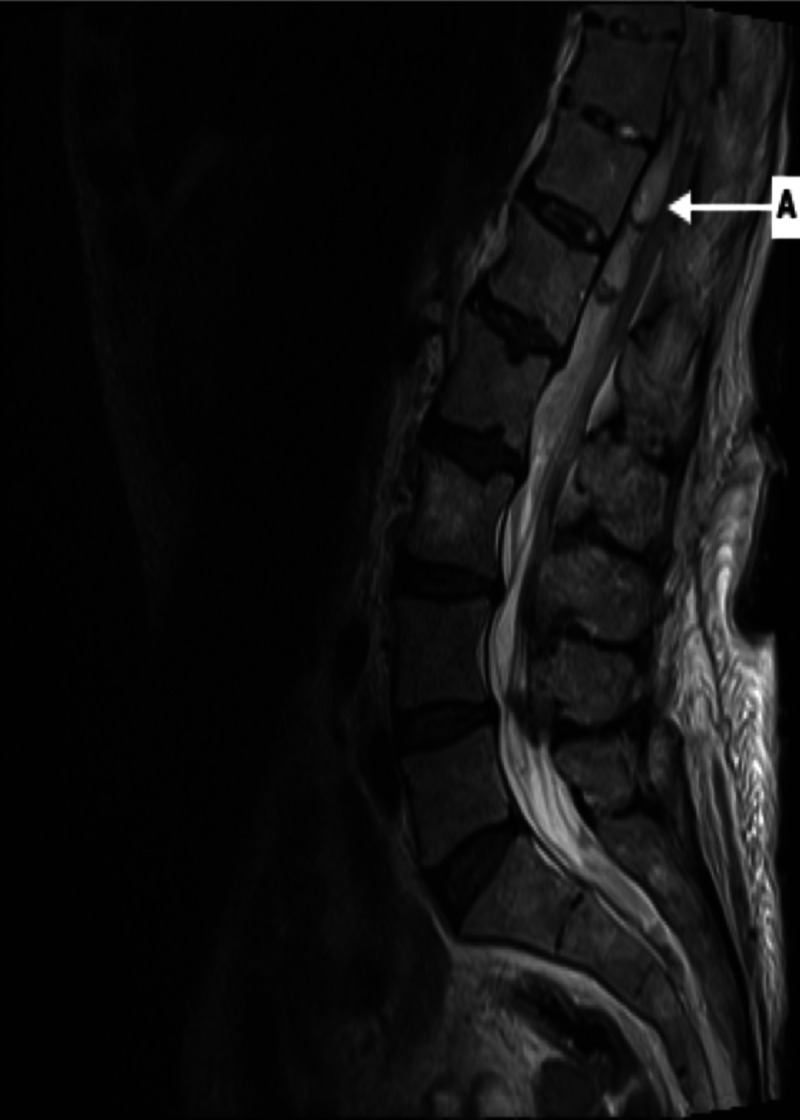
Magnetic resonance imaging T2 sagittal view Arrow A points to a heterogeneous collection at the anterior epidural space at T12 and L1 measuring 4.9 cm in length

**Figure 4:**
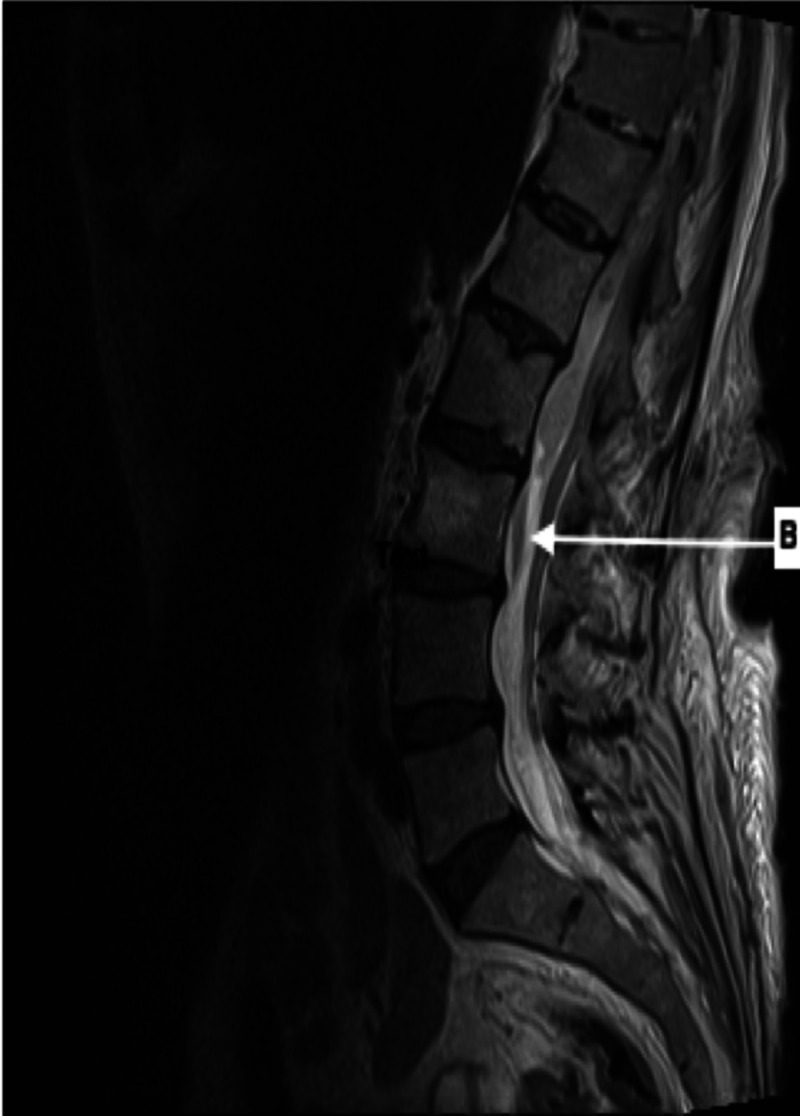
Magnetic resonance imaging T2 sagittal view Arrow B represents an additional area of heterogeneous collection posterior to L3 extending for 2.3 cm in length

Upon returning from the CT scan, the patient reported acute bilateral lower extremity motor weakness. A repeat musculoskeletal exam showed bilateral muscle strength 0/5 of both lower extremities, diminished sensation, and decreased Patellar and Achilles reflexes. Rectal exam revealed loose rectal tone. Patient was subsequently sent for an MRI with and without contrast of the thoracic and lumbar spine which revealed an anterior epidural hematoma versus abscess extending from T10 to L3 (Figures 3, 4).

Neurosurgery and intensive care were consulted. Neurosurgery recommended an immediate repeat MRI with and without contrast. Broad-spectrum antibiotics vancomycin and aztreonam were administered prior to obtaining the repeat MRI. Neurosurgery also recommended emergent evacuation of the hematoma with laminectomy as suggested time-to-surgery for SSEH is within 48 hours. Patient was given Vitamin K, 2 units of fresh frozen plasma, and Factor VII preoperatively due to lack of defined reversal strategies for rivaroxaban. The postoperative diagnosis was reported as a thoracic intradural extramedullary hematoma. Postoperatively, the patient was hypotensive and demonstrated atrial fibrillation with a rapid ventricular response. She was thus transferred to the ICU and managed with vasopressors, midodrine, and digoxin. Postoperative MRI revealed spinal cord infarction. Patient continued to be managed in-patient until her vital signs stabilized and were approved for discharge to home. In her six-week follow-up with the neurosurgeon, her surgical incision had healed; however, she had not yet regained motor or sensory function in either lower extremity.

## Discussion

The source of bleeding in spontaneous epidural hematomas may be from the rupture of the epidural veins, arteries, or due to vascular malformation. Arterial versus venous sources are often differentiated based on how soon and how complicated neurologic signs and symptoms present in the patient. Furthermore, the dorsal epidural space is more commonly affected by SSEHs and occur in areas of the spine with the most mobility, such as the cervicothoracic or thoracolumbar transition zones [4,10].

The gold standard imaging for a case of SSEH is MRI. If the clinical suspicion is confirmed, surgical decompression via laminectomy and irrigation and debridement is best done within 36 hours of symptom onset if complete sensorimotor loss is present and within 48 hours in the case of incomplete sensorimotor loss [1]. It is important to note that the patient’s neurologic status prior to the operative intervention is the most important prognostic factor for long-term outcome. Additionally, worst outcomes are associated when greater than or equal to four spinal segments are involved. If there are motor deficits but intact sensation, the prognosis improves [4,10].

In the case of our patient, she demonstrated rapid and progressive neurologic decline; thus, we postulate an arterial source [4,11]. The hematoma was also located in the ventral epidural space, which is rare, and extended within the thoracolumbar transition zone. Surgical decompression was done promptly within 48 hours from presentation. Complicated neurologic symptoms such as loss of motor function of both lower extremities presented just prior to surgery, thus predicting a poorer outcome. Lastly, her hematoma expanded across six spinal segments once again contributing to a poor prognosis. The patient’s use of rivaroxaban compounded her case as both a risk factor to developing SSEHs and a contributor to why her prognosis was poor.

## Conclusions

SSEHs are rare, progressive neurologic emergencies, which can present with non-specific lower back pain. Emergency physicians reportedly encounter 2.6 million annual emergency room visits for low back pain-related disorders, accounting for 2.3% of all ED visits annually in the US. Recent surveys show emergency physicians encounter patients with low back pain 5-20 times per month. Emergency physicians encounter patients with acute lower back pain regularly, as well as patients with atrial fibrillation. This case highlights the potential sequelae of non-specific, atraumatic lower back pain and how risk factors such as medications, including use of novel anticoagulants, can contribute to a devastating neurologic outcome.
